# Comparison of Cardiac Events Associated With Azithromycin vs Amoxicillin

**DOI:** 10.1001/jamanetworkopen.2020.16864

**Published:** 2020-09-15

**Authors:** Haridarshan Patel, Gregory S. Calip, Robert J. DiDomenico, Glen T. Schumock, Katie J. Suda, Todd A. Lee

**Affiliations:** 1 Department of Pharmacy Systems, Outcomes, and Policy, College of Pharmacy, University of Illinois at Chicago; 2 Flatiron Health, Inc, New York, New York; 3 Department of Pharmacy Practice, College of Pharmacy, University of Illinois at Chicago; 4 Center for Health Equity Research and Promotions, University of Pittsburgh School of Medicine, Pittsburgh, Pennsylvania; 5 Department of Medicine, Veterans Affairs Pittsburgh Healthcare System, Pittsburgh, Pennsylvania

## Abstract

**Importance:**

Conflicting evidence exists on the association between azithromycin use and cardiac events.

**Objective:**

To compare the odds of cardiac events among new users of azithromycin relative to new users of amoxicillin using real-world data.

**Design, Setting, and Participants:**

This retrospective cohort study used data from Truven Health Analytics MarketScan database from January 1, 2009, to June 30, 2015. Patients receiving either amoxicillin or azithromycin and enrolled in a health care plan 365 days before (baseline period) the dispensing date (index date) were included in the study. Patients were matched 1:1 on high-dimensional propensity scores. Data were analyzed from October 1, 2018, to December 31, 2019.

**Exposures:**

New use of azithromycin compared with new use of amoxicillin.

**Main Outcomes and Measures:**

The primary outcome consisted of cardiac events, including syncope, palpitations, ventricular arrhythmias, cardiac arrest, or death as a primary diagnosis for hospitalization at 5, 10, and 30 days from the index date. Logistic regression models were used to estimate odds ratios (ORs) with 95% CIs.

**Results:**

After matching, the final cohort included 2 141 285 episodes of each index therapy (N = 4 282 570) (mean [SD] age of patients, 35.7 [22.3] years; 52.6% female). Within 5 days after therapy initiation, 1474 cardiac events (0.03%) occurred (708 in the amoxicillin cohort and 766 in the azithromycin cohort). The 2 most frequent events were syncope (1032 [70.0%]) and palpitations (331 [22.5%]). The odds of cardiac events with azithromycin compared with amoxicillin were not significantly higher at 5 days (OR, 1.08; 95% CI, 0.98-1.20), 10 days (OR, 1.05; 95% CI, 0.97-1.15), and 30 days (OR, 0.98; 95% CI, 0.92-1.04). Among patients receiving any concurrent QT-prolonging drug, the odds of cardiac events with azithromycin were 1.40 (95% CI, 1.04-1.87) greater compared with amoxicillin. Among patients 65 years or older and those with a history of cardiovascular disease and other risk factors, no increased risk of cardiac events with azithromycin was noted.

**Conclusions and Relevance:**

This study found no association of cardiac events with azithromycin compared with amoxicillin except among patients using other QT-prolonging drugs concurrently. Although azithromycin is a safe therapy, clinicians should carefully consider its use among patients concurrently using other QT-prolonging drugs.

## Key Points

**Question:**

Using a large health care database, what is the risk of cardiac events in azithromycin users compared with amoxicillin users?

**Findings:**

In a cohort study of 2 141 285 episodes of azithromycin use that were matched to episodes of amoxicillin use with high-dimensional propensity scores, no difference in the rate of cardiac events occurred at 5, 10, and 30 days after therapy initiation. However, concurrent use of QT-prolonging drugs with azithromycin was associated with 40% greater odds of cardiac events compared with amoxicillin.

**Meaning:**

Azithromycin and amoxicillin appear to pose similar odds of cardiac events except for concurrent use of QT-prolonging drugs with azithromycin; therefore, clinicians should use amoxicillin or another antibiotic among such patients.

## Teaser

This observational cohort study compares the odds of cardiac events among new users of azithromycin relative to new users of amoxicillin using real-world data.

## Introduction

Azithromycin is a widely prescribed antibiotic. More than 30 million prescriptions of azithromycin are dispensed every year in the United States.^1^ Compared with other macrolide antibiotics that carry some adverse cardiac risks, azithromycin was considered safest because of its lower arrhythmogenic activity.^2^

More than 10 years after the drug’s approval in the United States, an early signal from case reports described QT interval prolongation, torsades de pointes (TdP), and ventricular tachycardia after the use of azithromycin.^3^ Since then, several retrospective, observational studies have investigated the risk of cardiac events with outpatient azithromycin use. In 2012, a study by Ray et al^4^ found a 2.9-fold higher risk of cardiac deaths within 5 days of an initial dispensing of azithromycin compared with amoxicillin. The risk was higher among patients with a history of cardiovascular disease. As a result, the US Food and Drug Administration (FDA) issued a warning to caution prescribing azithromycin in patients with known risk factors, such as existing QT interval prolongation, TdP, electrolyte abnormalities, uncompensated heart failure, bradycardia, and concomitant use of drugs that prolong the QT interval.^5^ Since then, 4 more studies^6,7,8,9^ have examined the cardiac risks with azithromycin by measuring cardiac deaths, cardiac events such as ventricular arrhythmias, or both. Three of 5 studies^4,6,7,8,9^ found an increased risk of deaths or arrhythmias with azithromycin. Two meta-analyses^10,11^ evaluating evidence from randomized clinical trials and observational studies found no significant increase in cardiac events. In a recent study,^1^ we showed that the prevalence of cardiac risk factors among azithromycin users remained similar before and after the FDA warning. Thus, despite the FDA warning, prescribing practices do not seem to have been modified. This outcome may be owing to the inconsistency in the results from the previous studies, which indicates a need to continue to evaluate this potential association.

Our present objective was to investigate the association of cardiac events among patients treated with azithromycin and amoxicillin using an active-control, new-user design. Moreover, we sought to evaluate the risk in prespecified subgroups that include elderly patients with cardiovascular disease, patients using concurrent QT-prolonging drugs before and after the FDA warning from May 2012, and the presence of risk factors identified from previous studies.^6,7,8,9^

## Methods

This cohort study was a population-level analysis of nationally representative databases of prescriptions dispensed from outpatient pharmacies in the United States. We followed the Strengthening the Reporting of Observational Studies in Epidemiology (STROBE) reporting guideline. The University of Illinois at Chicago investigational review board deemed that this study was exempt from review and informed consent owing to use of deidentified patient data. Patients prescribed azithromycin or amoxicillin for an acute bacterial infection from outpatient settings were included.

### Data Sources

We used data from the Truven Health Analytics MarketScan database,^12^ a private sector health data resource that reflects the health care experience of enrollees covered by the health benefit programs for employers. Data are collected from more than 100 different health insurance companies and are nationally representative of commercially insured enrollees and their dependents as well as Medicare-eligible beneficiaries with employer-provided Medicare supplemental plans.

### Study Cohort

We identified patients who were dispensed 1 or more azithromycin or amoxicillin prescriptions from January 1, 2009, through June 30, 2015. Multiple short-term dispensings (at least 30 days apart) of an index therapy for a single patient during the study period were treated as unique episodes. Based on the possible mechanism of action, the risk of cardiac events with azithromycin is expected to be acute and transient; thus, any subsequent exposure to the drug would still pose a similar risk regardless of prior exposures. Episodes were included if the patient had at least 365 days of continuous enrollment in a health plan (baseline period) before the dispense date of 1 index medication (index date). Episodes were excluded if the patient had a missing enrollment identification; if the patient was exposed to amoxicillin, azithromycin, clarithromycin, and erythromycin in the 30 days before and 5 days after the index date (follow-up period); or if the supply of the index therapy was greater than 14 days (as possible maintenance therapy).

### Follow-up Period

Patients were followed up to 5, 10, and 30 days after the index date. Patients were censored if they disenrolled from the health plan or at the end of the follow-up period, whichever occurred first.

### Covariates

Patient characteristics, including age, sex, geographic region, and insurance type, were collected at the index date. Baseline clinical conditions were categorized using Clinical Classifications Software.^13^ The software collapses diagnosis and procedure codes from the *International Classification of Diseases, Ninth Revision, Clinical Modification* (*ICD-9-CM*), which contains more than 14 000 diagnosis codes and 3900 procedure codes.

Drugs that prolong the QT interval or induce TdP were identified from a publicly available, well-established, and widely recognized list from the CredibleMeds website (see eTable 1 in the Supplement for the full list).^14^ The drugs were categorized into 24 different therapeutic classes and into known, possible, and conditional risk groups. Any QT-prolonging drugs prescribed within 90 days before the index date were considered. Concurrent use was defined based on an overlap of the days of supply (with a 15% allowed gap^15^) of QT-prolonging drugs with the index date of the azithromycin or amoxicillin prescription.

### Study End Point

The outcome was measured as a primary diagnosis during a hospitalization or first-listed emergency department visit based on *ICD-9-CM* codes for ventricular arrhythmia, palpitation, long QT syndrome, cardiac arrest, syncope, or death (codes 426.82, 427, 427.1, 427.2, 427.4, 427.5, 427.9, 427.41, 427.42, 780.2, 785.1, 798, 798.1, 798.2, or 798.9). This outcome definition was validated in previous studies with positive predictive values greater than 80%.^16,17^ We also applied an algorithm developed by another validation study to identify the outcomes originating from outpatient settings.^18^

### Propensity Score

We used high-dimensional propensity scores to control for any potential confounding between cohorts.^19,20^ Propensity scores were generated using a semiautomated approach in which 500 covariates were empirically selected across domains of inpatient and outpatient diagnosis, procedures, and drug dispensing. The covariates were ranked in order of their magnitude of association with the exposure. Furthermore, we included a prespecified list of the following variables in the final-exposure propensity score model: age, sex, calendar year, concurrent use of QT-prolonging drugs, and previously identified factors associated with cardiac events with azithromycin use (history of syncope, cardiac dysrhythmias, nonspecific chest pain, and use of antiarrhythmic agents, antiemetics, antidepressants, loop diuretics, and angiotensin-converting enzyme inhibitors). We compared models with and without the prespecified variables by examining the concordance statistic. We also assessed for multicollinearity by checking the variance inflation factor for each prespecified variable. We matched the cohorts 1:1 on high-dimensional propensity scores using the nearest neighbor matching approach with a caliper width of 0.2 SDs of the logit of the high-dimensional propensity score.^21^ We then assessed the balance of characteristics between cohorts using standardized differences (>10% was considered a significant difference between the groups).^22^ The eFigure in the Supplement shows the distribution of high-dimensional propensity scores between before matching and after matching.

### Subgroups

We examined an a priori list of subgroups to determine whether the risk of cardiac events between azithromycin and amoxicillin varied within these groups. We evaluated the risk among the subgroups of elderly patients (aged ≥65 years); patients with a history of syncope, cardiac dysrhythmias, or nonspecific chest pain; and patients with concurrent use of QT-prolonging drugs. A separate subgroup included patients with specific concomitant use of QT-prolonging medicines, including antiarrhythmic agents, antiemetics, antidepressants, loop diuretics, or angiotensin-converting enzyme inhibitors. Last, we examined the risk among patients with and without baseline cardiovascular disease. For each subgroup, the cohorts of patients receiving azithromycin and amoxicillin were matched based on their propensity scores.

### Statistical Analysis

Data analysis was performed from October 1, 2018, to December 31, 2019. For efficiency, we used a random sample of 25% of the total cohort to generate high-dimensional propensity scores. The unit of analysis consisted of episodes of index therapies. We used the logistic regression model to examine the association of cardiac risks with azithromycin and amoxicillin. We generated odds ratios (ORs) with 95% CIs, with amoxicillin as the reference cohort. We conducted several sensitivity analyses to evaluate the impact on study findings. First, we restricted our outcome definition to cardiac events identified only from inpatient or emergency department visits. Second, instead of using episodes or multiple dispensing of index therapies as our unit of analysis, we evaluated risk among unique patients by examining their first dispensing of index therapy. Third, we examined the risk before and after the FDA warning from 2012. Fourth, we evaluated the outcome at both 10 and 30 days. Fifth, we relaxed our exclusion criterion of a supply of index therapy of more than 14 days. To address the issue of multiple comparisons (n = 13), we applied the Benjamini-Hochberg procedure and presented the findings in eTable 3 in the Supplement. All statistical analyses were completed using SAS Enterprise Guide, version 7.1 (SAS Institute Inc). Two-sided *P* < .05 indicated significance.

## Results

The original cohort included more than 44 million episodes of azithromycin and amoxicillin use. After taking a random sample of 25%, 9 507 450 users of azithromycin and amoxicillin (25% of each cohort) were eligible for inclusion. After matching with high-dimensional propensity scores at a 1:1 ratio, the final cohort included 2 141 285 episodes of each index therapy (N = 4 282 570). The combined cohorts included episodes of patients with a mean (SD) age of 35.7 (22.3) years, of whom 52.6% were female and 47.4% were male (Table 1). Except for the preferred provider organization insurance category (standardized difference, 0.18 [>10%]), all of the baseline characteristics were similar between the amoxicillin and azithromycin cohorts. The duration of therapy was 5 days for 87.3% of azithromycin episodes and less than 10 days for 93.2% of amoxicillin episodes.

**Table 1.** Patient Characteristics

Characteristic	No. (%) of episodes^a^			Standardized difference
	All (N = 4 282 570)	Amoxicillin cohort (n = 2 141 285)	Azithromycin cohort (n = 2 141 285)	
Age group, y				
≤17	1 235 866 (28.9)	617 894 (28.9)	617 972 (28.9)	<0.01
18-34	768 295 (17.9)	383 856 (17.9)	384 439 (18.0)	<0.01
35-44	598 880 (14.0)	299 864 (14.0)	299 016 (14.0)	<0.01
45-54	651 738 (15.2)	325 719 (15.2)	326 019 (15.2)	<0.01
55-64	654 906 (15.3)	328 086 (15.3)	326 820 (15.3)	<0.01
≥65	372 885 (8.7)	185 866 (8.7)	187 019 (8.7)	<0.01
Sex				
Male	2 031 585 (47.4)	1 013 124 (47.3)	1 018 461 (47.6)	0.01
Female	2 250 985 (52.6)	1 128 161 (52.7)	1 122 824 (52.4)	0.01
Region				
Northeast	903 860 (21.1)	461 705 (21.6)	442 155 (20.6)	0.03
North Central	999 561 (23.3)	500 051 (23.4)	499 510 (23.3)	<0.01
South	1 410 701 (32.9)	683 762 (31.9)	726 939 (33.9)	0.04
West	822 314 (19.2)	415 616 (19.4)	406 698 (19.0)	0.01
Unknown	146 134 (3.4)	80 151 (3.7)	65 983 (3.1)	0.03
Insurance type				
PPO	1 689 493 (39.5)	749 262 (35.0)	940 231 (43.9)	0.18
HMO	769 470 (18.0)	394 198 (18.4)	375 272 (17.5)	0.02
Point-of-service plan	429 326 (10.0)	245 686 (11.5)	183 640 (8.6)	0.10
Consumer-directed health plan	469 696 (11.0)	246 362 (11.5)	223 334 (10.4)	0.04
Comprehensive coverage	280 511 (6.6)	145 879 (6.8)	134 632 (6.3)	0.02
High-deductible health plan	301 894 (7.0)	169 706 (7.9)	132 188 (6.2)	0.07
EPO	114 126 (2.7)	65 353 (3.1)	48 773 (2.3)	0.05
Other	46 505 (1.1)	28 548 (1.3)	17 957 (0.8)	0.05
Missing or unknown	181 549 (4.2)	96 291 (4.5)	85 258 (4.0)	0.03
Year				
2010	569 017 (13.3)	284 864 (13.3)	284 153 (13.3)	<0.01
2011	719 032 (16.8)	357 468 (16.7)	361 564 (16.9)	0.01
2012	803 540 (18.8)	390 664 (18.2)	412 876 (19.3)	0.03
2013	775 556 (18.1)	389 490 (18.2)	386 066 (18.0)	0.01
2014	876 488 (20.5)	442 403 (20.7)	434 085 (20.3)	0.01
2015	538 937 (12.6)	276 396 (12.9)	262 541 (12.3)	0.02

Abbreviations: EPO, exclusive provider organization; HMO, health maintenance organization; PPO, preferred provider organization.

^a^ Percentages have been rounded and may not total 100.

More than one-third of episodes (34.5%) included patients with a history of disease of the circulatory systems (Table 2). Among such episodes, 20.8% had hypertension, 11.1% had nonspecific chest pain, and 7.3% had cardiac dysrhythmias. More than one-half of episodes (53.5%) included patients with a history of respiratory infection, and almost one-quarter (23.7%) included patients with a mental illness. The prevalence of these conditions was similar between azithromycin and amoxicillin users. More than one-fifth of episodes (20.4%) included at least 1 concurrent use of a QT-prolonging drug, and 6.6% used at least 2 concurrent drugs with the index therapies (Table 3). Antidepressants (7.3%) and opiate agonists (2.3%) were the most commonly prescribed drugs. The use of at least 1 QT-prolonging drug with known risk was 6.0%; with possible risk, 5.5%; and with conditional risk, 12.8%. The use of concurrent QT-prolonging drugs was similar between the amoxicillin and azithromycin cohorts.

**Table 2.** Comorbid Conditions

Condition	No. (%) of episodes			Standardized difference
	All (N = 4 282 570)	Amoxicillin cohort (n = 2 141 285)	Azithromycin cohort (n = 2 141 285)	
Viral infection	665 825 (15.5)	337 233 (15.7)	328 592 (15.3)	0.01
Diseases of the genitourinary system	1 470 951 (34.3)	728 193 (34.0)	742 758 (34.7)	0.01
Urinary tract infections	445 235 (10.4)	224 384 (10.5)	220 851 (10.3)	0.01
Diseases of the skin and subcutaneous tissue	1 271 184 (29.7)	621 489 (29.0)	649 695 (30.3)	0.03
Skin and subcutaneous tissue infections	370 416 (8.6)	189 142 (8.8)	181 274 (8.5)	0.01
Cellulitis and abscess	290 183 (6.8)	147 908 (6.9)	142 275 (6.6)	0.01
Diseases of the musculoskeletal system and connective tissue	1 940 221 (45.3)	937 496 (43.8)	1 002 725 (46.8)	0.06
Neoplasms	960 376 (22.4)	465 600 (21.7)	494 776 (23.1)	0.03
Endocrine, nutritional, and metabolic diseases and immunity disorders	1 701 472 (39.7)	839 661 (39.2)	861 811 (40.2)	0.02
Diseases of the blood and blood-forming organs	366 401 (8.6)	182 480 (8.5)	183 921 (8.6)	<0.01
Mental illness	1 015 308 (23.7)	499 874 (23.3)	515 434 (24.1)	0.02
Diseases of the circulatory system	1 477 867 (34.5)	725 470 (33.9)	752 397 (35.1)	0.03
Hypertension	891 160 (20.8)	440 414 (20.6)	450 746 (21.1)	0.01
Nonspecific chest pain	473 800 (11.1)	226 797 (10.6)	247 003 (11.5)	0.03
Cardiac dysrhythmias	311 938 (7.3)	153 810 (7.2)	158 128 (7.4)	0.01
Diseases of the respiratory system	2 750 481 (64.2)	1 338 330 (62.5)	1 412 151 (65.9)	0.07
Respiratory infections	2 289 618 (53.5)	1 116 824 (52.2)	1 172 794 (54.8)	0.05
Acute bronchitis	550 876 (12.9)	259 620 (12.1)	291 256 (13.6)	0.04
Acute upper respiratory tract infections	935 848 (21.9)	464 338 (21.7)	471 510 (22.0)	0.01
Chronic sinusitis	338 836 (7.9)	166 964 (7.8)	171 872 (8.0)	0.01
Chronic obstructive pulmonary disease	297 563 (6.9)	134 693 (6.3)	162 870 (7.6)	0.05
Asthma	439 495 (10.3)	190 550 (8.9)	248 945 (11.6)	0.09
Diseases of the digestive system	1 313 916 (30.7)	658 930 (30.8)	654 986 (30.6)	<0.01

**Table 3.** Concurrent Use of QT-Prolonging Drugs

Drug	No. (%) of episodes			Standardized difference
	All (N = 4 282 570)	Amoxicillin cohort (n = 2 141 285)	Azithromycin cohort (n = 2 141 285)	
Antidepressants	312 772 (7.3)	153 054 (7.1)	159 718 (7.5)	0.01
Opiate agonists	96 447 (2.3)	52 119 (2.4)	44 328 (2.1)	0.02
Diuretics	79 861 (1.9)	39 992 (1.9)	39 869 (1.9)	<0.01
Gastrointestinal tract drugs	64 209 (1.5)	30 836 (1.4)	33 373 (1.6)	0.01
ACE inhibitors	62 652 (1.5)	32 011 (1.5)	30 641 (1.4)	0.01
Loop diuretics	59 753 (1.4)	29 830 (1.4)	29 923 (1.4)	<0.01
Quinolones	53 595 (1.3)	20 094 (0.9)	33 501 (1.6)	0.06
Antitussives and cold combination	39 417 (0.9)	10 840 (0.5)	28 577 (1.3)	0.09
Analgesics and antipyretics	35 777 (0.8)	18 544 (0.9)	17 233 (0.8)	0.01
Antipsychotics	32 949 (0.8)	16 795 (0.8)	16 154 (0.8)	<0.01
Potassium-sparing diuretics	23 274 (0.5)	11 483 (0.5)	11 791 (0.6)	<0.01
Antiemetics	16 029 (0.4)	8059 (0.4)	7970 (0.4)	<0.01
Antimalarial agents	12 842 (0.3)	5940 (0.3)	6902 (0.3)	0.01
β-Blockers	12 710 (0.3)	6589 (0.3)	6121 (0.3)	<0.01
Antiarrhythmic agents	11 424 (0.3)	7026 (0.3)	4398 (0.2)	0.02
Histamine antagonists	10 607 (0.2)	5402 (0.3)	5205 (0.2)	<0.01
Muscle relaxers, skeletal central	9454 (0.2)	4646 (0.2)	4808 (0.2)	<0.01
Anxiolytics, sedatives, and/or hypnotics	8285 (0.2)	3964 (0.2)	4321 (0.2)	<0.01
Parasympathomimetics	8277 (0.2)	4062 (0.2)	4215 (0.2)	<0.01
Anti-infectives	7088 (0.2)	5300 (0.2)	1788 (0.1)	0.04
Total No. of drugs				
≥1	874 935 (20.4)	431 747 (20.2)	443 188 (20.7)	0.04
≥2	282 367 (6.6)	139 766 (6.5)	142 601 (6.7)	
Drugs with a known risk^a^				
≥1	255 474 (6.0)	128 809 (6.0)	126 665 (5.9)	<0.01
≥2	33 062 (0.8)	16 748 (0.8)	16 314 (0.8)	
Drugs with a possible risk^a^				
≥1	234 441 (5.5)	112 667 (5.3)	121 774 (5.7)	0.05
≥2	39 551 (0.9)	19 585 (0.9)	19 966 (0.9)	
Drugs with a conditional risk^a^				
≥1	546 415 (12.8)	270 602 (12.6)	275 813 (12.9)	<0.01
≥2	134 506 (3.1)	66 313 (3.1)	68 193 (3.2)	

Abbreviation: ACE, angiotensin-converting enzyme.

^a^ Defined based on CredibleMeds.org list.

Within 5 days after treatment initiation, the occurrence of cardiac events among the overall cohort was 0.03% (n = 1474), or 3.4 events per 10 000 episodes (Table 4). The most frequent diagnoses of cardiac events included syncope at 70.0% and palpitations at 22.5%. Seven hundred eight cardiac events occurred in amoxicillin users and 766 in azithromycin users within 5 days. The prevalence of cardiac events within 10 days was 4.9 events per 10 000 episodes; within 30 days, 10.0 events per 10 000 (eTable 2 in the Supplement).

**Table 4.** Occurrence of Cardiac Events

Event	No. (%) of episodes			*P* value
	Total (N = 4 282 570)	Amoxicillin cohort (n = 2 141 285)	Azithromycin cohort (n = 2 141 285)	
Within 5 d, all^a^	1474 (0.03)	708 (0.03)	766 (0.03)	.13
Syncope	1032 (70.0)	494 (69.8)	538 (70.2)	.18
Palpitations	331 (22.5)	156 (22.0)	175 (22.8)	.08
Cardiac dysrhythmia	61 (4.1)	31 (4.4)	30 (3.9)	>.99
Cardiac arrest	51 (3.5)	24 (3.4)	27 (3.5)	.67
Paroxysmal ventricular tachycardia	20 (1.4)	13 (1.8)	7 (0.9)	.90
Ventricular fibrillation	3 (0.2)	3 (0.4)	0	NA
Ventricular flutter	2 (0.1)	1 (0.1)	1 (0.1)	.30
Instantaneous death	1 (0.1)	1 (0.1)	0	.17
Long QT syndrome	0	0	0	.32
Within 10 d^b^	2054 (0.05)	998 (0.05)	1056 (0.05)	.20
Within 30 d^c^	4101 (0.1)	2072 (0.1)	2029 (0.1)	.50

Abbreviation: NA, not applicable.

^a^ Includes 4 282 570 episodes, with 2 141 285 in each cohort.

^b^ Includes 4 234 226 episodes, with 2 117 113 in each cohort. Individual events are listed in eTable 2 in the Supplement.

^c^ Includes 4 105 722 episodes, with 2 052 861 in each cohort. Individual events are listed in eTable 2 in the Supplement.

Compared with amoxicillin, the odds of cardiac events within 5 days with azithromycin were similar (OR, 1.08; 95% CI, 0.98-1.20) (Table 5). Similar findings were observed when extending the follow-up period to 10 (OR, 1.05; 95% CI, 0.97-1.15) and 30 (OR, 0.98; 95% CI, 0.92-1.04) days because there was no association between azithromycin use and cardiac events when compared with amoxicillin. In all subgroups, the rate of events was higher in the azithromycin group; however, none of the events were statistically significant. One exception was when the analysis was restricted to patients using QT-prolonging medications. In this subgroup, the azithromycin users had a 40% increased risk of cardiac events during the 5 days of follow-up when compared with amoxicillin users (OR, 1.40; 95% CI, 1.04-1.87). The results of the sensitivity analyses of unique patients before and after the FDA warning, cardiac events identified from inpatient diagnosis only, and inclusion of patients regardless of their supply duration were consistent with the findings from our base-case analysis.

**Table 5.** Outcomes Associated With Use of Azithromycin Compared With Amoxicillin

Variable	No. of episodes	OR (95% CI)^a^
Cardiac events in overall cohort		
Within 5 d	4 282 570	1.08 (0.98-1.20)
Within 10 d	4 234 226	1.05 (0.97-1.15)
Within 30 d	4 105 722	0.98 (0.92-1.04)
Subgroups		
Age ≥65 y	372 885	1.21 (0.96-1.52)
Concurrent use of QT-prolonging drugs	874 935	1.40 (1.04-1.87)
Factors associated with cardiac events		
Model 1^b^	1 318 514	1.15 (0.94-1.40)
Model 2^c^	434 233	1.29 (0.73-2.27)
Cardiovascular disease		
No	4 157 508	1.07 (0.96-1.19)
Yes	125 062	2.00 (0.75-5.33)
Sensitivity analyses		
Unique patients	3 423 819	1.11 (0.98-1.26)
Before the FDA warning from May 2012	1 659 140	1.08 (0.90-1.30)
After the FDA warning from May 2012	2 623 430	1.09 (0.96-1.25)
Cardiac events within 5 d^d^	4 282 261	1.11 (0.98-1.23)

Abbreviations: FDA, US Food and Drug Administration; OR, odds ratio.

^a^ Indicates odds of a cardiac event compared with use of amoxicillin (reference category).

^b^ Included age, sex, history of syncope, cardiac dysrhythmias, nonspecific chest pain, and concurrent use of QT-prolonging drug.

^c^ Included age, sex, antiarrhythmic agents, antiemetics, antidepressants, loop diuretics, and angiotensin-converting enzyme inhibitors.

^d^ Revised outcome definition based on inpatient diagnoses only.

## Discussion

This study contributes evidence to the association of cardiac events with azithromycin. Using real-world data from an extensive claims database, we found no increased odds of cardiac events with azithromycin within 5 days after treatment initiation, but a small, clinically relevant association was found among patients concurrently using a QT-prolonging drug. Elderly patients, those with a history of cardiovascular disease, and those with other baseline risk factors had numerically higher odds of cardiac events with azithromycin.

The evidence from previous studies has also suggested a small increase in the absolute risk of cardiac arrhythmias with azithromycin, but such risk, when compared with that of another antibiotic, is negligible. In 2017, Trifirò et al^9^ conducted a study using data from 7 European countries and found no increase in cardiac arrhythmias with azithromycin compared with amoxicillin. Our findings are consistent with those of Trifirò et al^9^ but not with those of 2 other studies that similarly examined the risk among the US Veterans Health Administration^6^ and Taiwanese cohorts.^7^ Compared with other studies, we included a younger cohort (on average, 10 years younger) and did not exclude patients based on their comorbid conditions to make our findings more generalizable. We also included the cardiac events syncope and tachycardia in our outcome definition because we strongly believe both conditions are intermediaries in the sequelae of cardiac arrhythmias. We also controlled for the previously identified factors associated with cardiac events among azithromycin users. Last, we used the high-dimensional propensity score method, whereas previous studies used a traditional propensity scoring method or matching to a control group for confounding. Such differences in cohort, definitions, and study methods could explain the inconsistencies in the findings between the studies.

More than 200 drugs are associated with prolonging the QT interval.^14^ Such drugs may confound the association between azithromycin and cardiac events. Thus, similar to the study by Trifirò et al,^9^ we controlled for the confounding effects of concurrent use of QT-prolonging drugs with azithromycin. In the subgroups, including episodes with concomitant use of QT-prolonging drugs, we found a significantly higher risk with azithromycin. Although we were unable to examine the risk of cardiac events by each therapeutic class of QT-prolonging drugs, we found that most episodes included patients who were prescribed QT-prolonging antidepressants. This finding suggests that cardiac risk may be modified by the presence of any QT-prolonging drug and not just antiarrhythmic drugs, as previously thought. In a sample of the cohort, we explored the likelihood of patients receiving a QT-prolonging medication with azithromycin vs amoxicillin. We found that patients were more likely to be prescribed QT-prolonging drugs with azithromycin. Although we were not able to ascertain the reasons for such findings, accounting for the use of QT-prolonging drugs when examining the risks associated with azithromycin may be necessary. We hypothesize that the confounding effect of other QT-prolonging drugs may help explain and resolve the inconsistent findings from previous studies.

### Strengths and Limitations

Our study has several strengths. Given a large cohort without many inclusion and exclusion criteria, our findings are likely to be generalizable to the entire population in the United States. We used a robust method and an extensive list of previously identified confounders to control for confounding.

Our findings are to be interpreted within the context of observational studies and their limitations. First, retrospective observational studies are always prone to unmeasured confounding and measurement error. Despite the large denominator, the power of the study was low. We used data from administrative health care claims, which are primarily used for billing and not research purposes. We were unable to ascertain essential risk factors, including race, smoking status, use of over-the-counter drugs, abnormal electrolyte levels, recent QT-interval prolongation, or body mass index because these variables were not available in the database. Because we could not measure these factors, we used high-dimensional propensity scores in our study such that proxies of these unmeasured confounders might be included, resulting in balance across the exposure groups. Although we were able to measure the outcome of death using *ICD-9-CM* codes, we know this would underestimate the number of deaths because we lacked information on death occurring in settings other than during an inpatient hospitalization. From our database, we did not have access to the reasons for death (cardiac vs no cardiac). As a result, our study findings should be interpreted in the context of risk of cardiac events and not death.

## Conclusions

In this large cohort study including more than 4 million episodes of both azithromycin and amoxicillin use, we found no increased odds of cardiac events with azithromycin in the overall cohort but a significantly higher odds among patients prescribed concurrent QT-prolonging drugs with azithromycin. Alternative antibiotic therapies should be considered among these patients.

## Notes

Supplement. **eTable 1.** List of QT-Prolonging Drugs From CredibleMeds.Org **eTable 2.** Frequency of Cardiac Events **eTable 3.** Benjamini-Hochberg Procedure **eFigure.** Distribution of High-Dimensional Propensity Score Between Cohorts
