# Supplementary material for: Comparison of Cardiac Events Associated With Azithromycin vs Amoxicillin
Source: JAMA Netw Open. 2020 Sep 15;3(9):e2016864. doi: 10.1001/jamanetworkopen.2020.16864 (PMC7492910; doi:10.1001/jamanetworkopen.2020.16864)
Supplement: Supplement. — eTable 1. List of QT-Prolonging Drugs From CredibleMeds.Org eTable 2. Frequency of Cardiac Events eTable 3. Benjamini-Hochberg Procedure eFigure. Distribution of High-Dimensional Propensity Score Between Cohorts [file jamanetwopen-e2016864-s001.pdf]

Supplementary Online Content

Patel H, Calip GS, DiDomenico RJ, Schumock GT, Suda KJ, Lee TA. Comparison of cardiac events associated with azithromycin vs amoxicillin. *JAMA Netw Open*. 2020;3(9):e2016864. doi:10.1001/jamanetworkopen.2020.16864

eTable 1. List of QT-Prolonging Drugs From CredibleMeds.Org

eTable 2. Frequency of Cardiac Events

eTable 3. Benjamini-Hochberg Procedure

eFigure. Distribution of High-Dimensional Propensity Score Between Cohorts

This supplementary material has been provided by the authors to give readers additional information about their work.

©2020 Patel H et al. *JAMA Network Open*.

eTable 1. List of QT-Prolonging Drugs From CredibleMeds.Org

**Drugs**
Antidepressants
Desvenlafaxine
Maprotiline Hydrochloride
Paroxetine Mesylate
Amitriptyline Hydrochloride/chlordiazepoxide
Amitriptyline Hydrochloride/perphenazine
Desipramine Hydrochloride
Fluvoxamine Maleate
Imipramine Hydrochloride
Fluoxetine Hydrochloride
Venlafaxine Hydrochloride
Mirtazapine
Desvenlafaxine Succinate
Amitriptyline Hydrochloride
Paroxetine Hydrochloride
Trazodone Hydrochloride
Escitalopram Oxalate
Citalopram Hydrobromide
Sertraline Hydrochloride
Opiate Agonists
Hydrocodone Bitartrate
Hydrocodone Bitartrate/ibuprofen
Methadone Hydrochloride
Acetaminophen/hydrocodone Bitartrate
Cardiac Drugs
Ivabradine
Aliskiren/amlodipine Besylate/hydrochlorothiazide
Eprosartan Mesylate/hydrochlorothiazide
Aliskiren/hydrochlorothiazide
Candesartan Cilexetil/hydrochlorothiazide
Ranolazine

©2020 Patel H et al. *JAMA Network Open*.

Hydrochlorothiazide/irbesartan
Hydrochlorothiazide/telmisartan
Hydrochlorothiazide/olmesartan Medoxomil
Hydrochlorothiazide/valsartan
Hydrochlorothiazide/losartan Potassium
Diuretics, Thiazides
Metolazone
Indapamide
Hydrochlorothiazide
Gastrointestinal Drugs
Omeprazole
Lansoprazole
Pantoprazole Sodium
ACE Inhibitors
Captopril/hydrochlorothiazide
Fosinopril Sodium/hydrochlorothiazide
Moexipril Hydrochloride
Hydrochlorothiazide/moexipril Hydrochloride
Hydrochlorothiazide/quinapril Hydrochloride
Enalapril Maleate/hydrochlorothiazide
Benazepril Hydrochloride/hydrochlorothiazide
Hydrochlorothiazide/lisinopril
Diuretics, Loop Diuretics
Furosemide
Analgesics/Antipyretics
Acetaminophen/tramadol Hydrochloride
Tramadol Hydrochloride
Antipsychotics
Iloperidone
Olanzapine Pamoate
Haloperidol Decanoate
Paliperidone Palmitate
Pimozide
Iloperidone
Thioridazine Hydrochloride
Clozapine
Chlorpromazine Hydrochloride

©2020 Patel H et al. *JAMA Network Open*.

Paliperidone
Perphenazine
Asenapine
Haloperidol
Olanzapine
Risperidone
Aripiprazole
Quetiapine Fumarate
Diuretics, Potassium-Sparing
Amiloride Hydrochloride/hydrochlorothiazide
Hydrochlorothiazide/spironolactone
Hydrochlorothiazide/triamterene
Diphenhydramine Hydrochloride
Dextromethorphan Polistirex
Homatropine Methylbromide/hydrocodone Bitartrate
Chlorpheniramine Polistirex/hydrocodone Polistirex
Antimalarial Agents
Artemether/lumefantrine
Primaquine Phosphate
Chloroquine Phosphate
Hydroxychloroquine Sulfate
Antiemetics
Dolasetron Mesylate
Granisetron Hydrochloride
Ondansetron
Metoclopramide Hydrochloride
Ondansetron Hydrochloride
Beta Blockers
Hydrochlorothiazide/metoprolol Succinate
Hydrochlorothiazide/propranolol Hydrochloride
Bendroflumethiazide/nadolol
Hydrochlorothiazide/metoprolol Tartrate
Sotalol Hydrochloride
Bisoprolol Fumarate/hydrochlorothiazide
Antifungal
Amphotericin B Lipid Complex
Itraconazole

©2020 Patel H et al. *JAMA Network Open*.

Posaconazole
Voriconazole
Ketoconazole
Fluconazole
Muscle Relaxants
Tizanidine Hydrochloride
Tolterodine Tartrate
Mirabegron
Solifenacin Succinate
Histamine (H2) Antagonists
Calcium Carbonate/famotidine/magnesium Hydroxide
Cimetidine
Famotidine
Antiarrhythmic Agents
Quinidine Sulfate
Quinidine Gluconate
Propafenone Hydrochloride
Dronedarone Hydrochloride
Flecainide Acetate
Amiodarone Hydrochloride
Anxiolytic/Sedative/Hypnotic
Doxepin Hydrochloride
Hydroxyzine Hydrochloride
Parasympathomimetic
Galantamine Hydrobromide
Donepezil Hydrochloride
Antihistamines & Comb
Diphenhydramine Tannate/phenylephrine Tannate
Generic Drug Name
Diphenhydramine Hydrochloride
Brompheniramine Mal/diphenhydramine Hcl
Promethazine Hydrochloride
Hormone-Modifying Therapy
Toremifene Citrate
Leuprolide Acetate
Tamoxifen Citrate
CNS Agents, Misc.

©2020 Patel H et al. *JAMA Network Open*.

Memantine Hydrochloride;memantine Hydrochloride
Tetrabenazine
Memantine Hydrochloride
Ainfectives, Misc
Metronidazole
Unclassified Agents
Alfuzosin Hydrochloride
Antivirals
Atazanavir/cobicistat
Rilpivirine Hydrochloride
Saquinavir Mesylate
Telaprevir
Nelfinavir Mesylate
Amantadine Hydrochloride
Efavirenz
Emtricitabine/rilpivirine Hydrochloride/tenofovir
Lopinavir/ritonavir
Efavirenz/emtricitabine/tenofovir Disoproxil Fum
Antiplatelet Agents
Cilostazol
Antimanic Agents
Lithium Carbonate
Cardiac, Calcium Channel
Isradipine
Amlodipine Besylate/hydrochlorothiazide/valsartan
Anal/Antipyr, Opiate Part Agonist
Buprenorphine Hydrochloride
Buprenorphine/naloxone
Phosphodiesterase Inhibitors
Vardenafil Hydrochloride
Analg/Antipyr, Nonsteroid/Antiinflam
Famotidine/ibuprofen
Esomeprazole Magnesium/naproxen
Chemotherapy
Capecitabine
Molecular Targeted Therapy
Bosutinib

©2020 Patel H et al. *JAMA Network Open*.

Vemurafenib
Pazopanib Hydrochloride
Lapatinib Ditosylate
Dasatinib
Anticonvulsants
Ezogabine
Felbamate
Cough/Cough/Cold Comb
Dm Hydrobrom/diphenhydramine Hcl/phenyleph Hcl
Dextromethorphan Hydrobromide/guaifenesin
Diphenhydramine Hcl/pse Hcl
Interferons, Antineoplastic
Vandetanib
Sorafenib Tosylate
Antitussives
Diphenhydramine Hydrochloride
Dextromethorphan Polistirex
Homatropine Methylbromide/hydrocodone Bitartrate
Chlorpheniramine Polistirex/hydrocodone Polistirex

©2020 Patel H et al. *JAMA Network Open*.

eTable 2. Frequency of Cardiac Events

	Total	Amoxicillin	Azithromycin	P-value
**Outcome within 5 days**	N=4,282,570	N=2,141,285	N=2,141,285	
**Overall**	0.0%	0.0%	0.0%	0.131
Syncope	70.0%	69.8%	70.2%	0.180
Palpitations	22.5%	22.0%	22.9%	0.083
Cardiac dysrhythmia	4.1%	4.4%	3.9%	1.000
Cardiac arrest	3.5%	3.4%	3.5%	0.674
Paroxysmal ventricular tachycardia	1.4%	1.8%	0.9%	0.898
Ventricular fibrillation	0.2%	0.4%	0.0%	-
Ventricular flutter	0.1%	0.1%	0.1%	0.296
Instantaneous death	0.1%	0.1%	0.0%	0.171
Long QT syndrome	0.0%	0.0%	0.0%	0.317
**Outcome within 10 days**	N=4,234,226	N=2,117,113	N=2,117,113	
**Overall**	0.05%	0.05%	0.05%	0.201
Syncope	68.2%	68.7%	67.6%	0.454
Palpitations	22.9%	22.0%	23.7%	0.166
Cardiac arrest	4.3%	4.2%	4.5%	0.596
Cardiac dysrhythmia	4.0%	4.2%	3.8%	0.825
Paroxysmal ventricular tachycardia	1.9%	2.0%	1.7%	0.746
Ventricular fibrillation	0.3%	0.5%	0.1%	0.103

©2020 Patel H et al. *JAMA Network Open*.

Ventricular flutter	0.2%	0.1%	0.2%	0.564
Instantaneous death	0.1%	0.2%	0.0%	0.157
Long QT syndrome	0.0%	0.0%	0.0%	-
**Outcome within 30 days**	N=4,105,722	N=2,052,861	N=2,052,861	
**Overall**	0.1%	0.1%	0.1%	0.502
Syncope	64.8%	64.0%	65.6%	0.938
Palpitations	25.7%	25.8%	25.5%	0.579
Cardiac arrest	4.9%	5.0%	4.7%	0.620
Cardiac dysrhythmia	4.1%	4.4%	3.8%	0.246
Paroxysmal ventricular tachycardia	2.4%	2.6%	2.2%	0.312
Ventricular fibrillation	0.4%	0.6%	0.3%	0.059
Ventricular flutter	0.2%	0.1%	0.3%	0.103
Long QT syndrome	0.0%	0.0%	0.0%	-
Instantaneous death	0.1%	0.1%	0.0%	0.083

©2020 Patel H et al. *JAMA Network Open*.

eTable 3. Benjamini-Hochberg Procedure

	**P-values**	**Rank**	**(I/M)Q**
Concurrent QT-prolonging drugs	0.0269	1	0.0192
**≥65 years old**	**0.1042**	**2**	**0.0385**
Outcomes (only from inpatient claims)	0.1102	3	0.0577
Within 5 days	0.1310	4	0.0769
With Cardiovascular disease	0.1657	5	0.0962
Predictors from Model 1	0.1714	6	0.1154
After the FDA warning	0.1850	7	0.1346
Within 10 days	0.2007	8	0.1538
Without cardiovascular disease	0.2210	9	0.1731
Predictors from Model 2	0.3877	10	0.1923
Before the FDA warning	0.4035	11	0.2115
Unique Patients	0.4539	12	0.2308
Within 30 days	0.5017	13	0.2500

©2020 Patel H et al. *JAMA Network Open*.

eFigure. Distribution of High-Dimensional Propensity Score Between Cohorts

A. Before matching

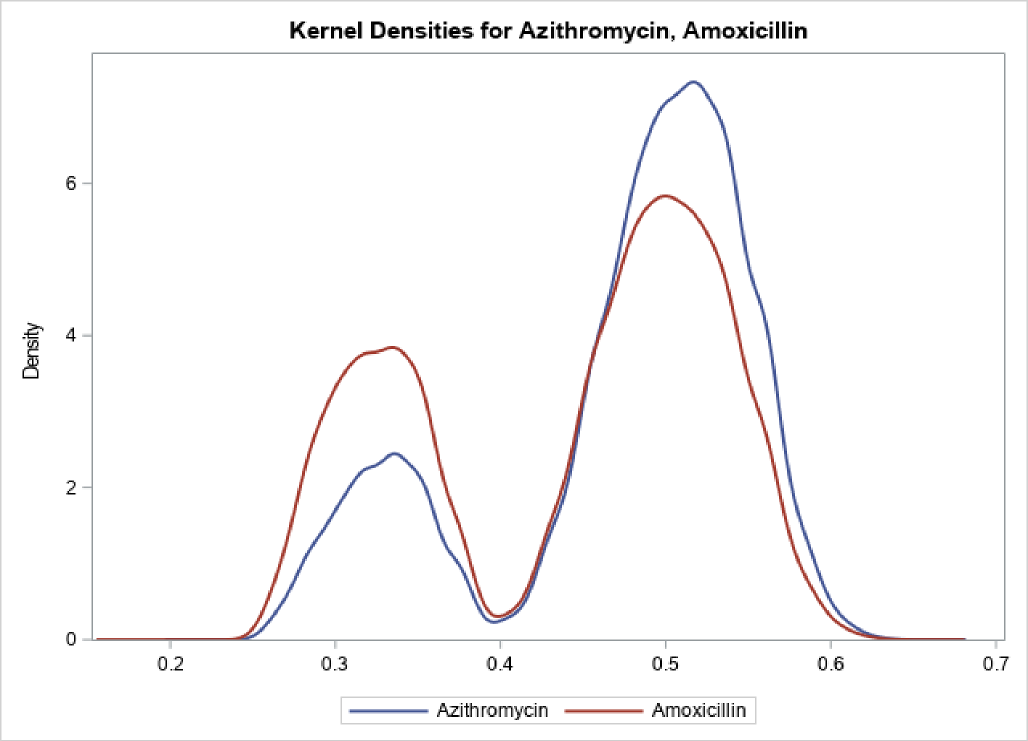

B. After matching

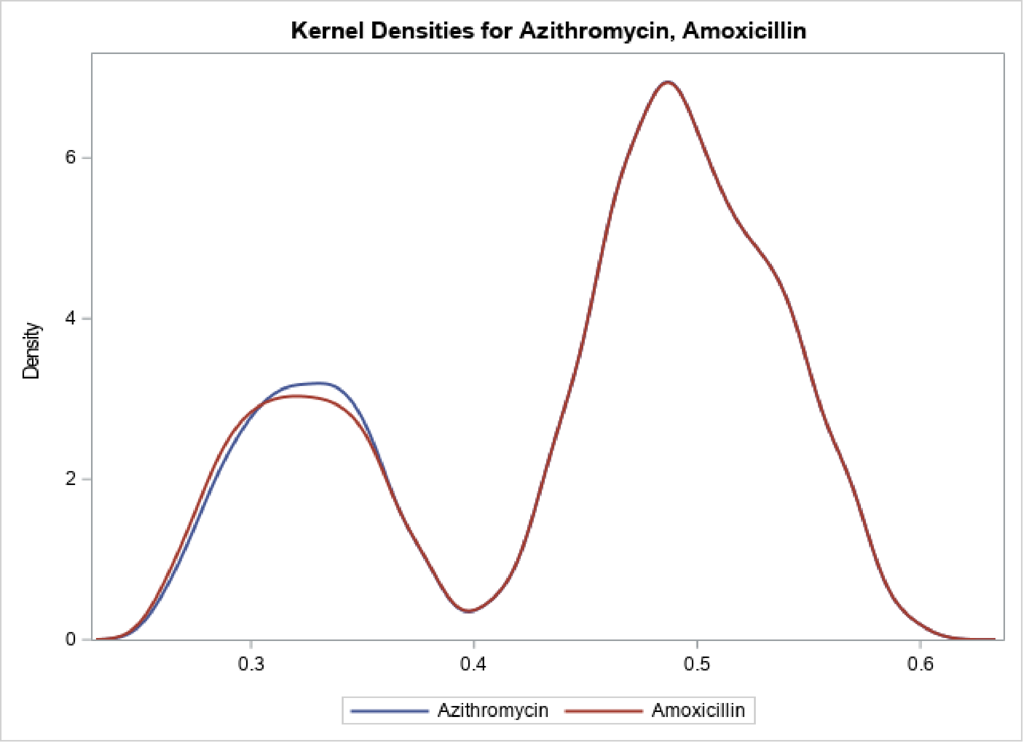

©2020 Patel H et al. *JAMA Network Open*.
